# Indigenous maternal health and health services within Canada: a scoping review

**DOI:** 10.1186/s12884-023-05645-y

**Published:** 2023-05-08

**Authors:** Meagan Bacciaglia, Hannah Tait Neufeld, Elena Neiterman, Akanksha Krishnan, Sophie Johnston, Kyla Wright

**Affiliations:** 1 School of Public Health Sciences, The University of Waterloo, Waterloo, ON Canada; 2 Faculty of Arts, Wilfrid Laurier University, Waterloo, ON Canada

**Keywords:** Maternal health, Cultural sensitivity, Indigenous, Healthcare delivery, Health inequalities, Pregnancy, Health education, Prenatal care, Canada, Scoping review

## Abstract

**Background:**

Globally, there are disparities in access to maternal health care services and equity in maternal health outcomes between Indigenous and non-Indigenous populations. While the literature is growing, it has not been systematically synthesized. This review addresses this gap by synthesizing the existing literature on the organizational structure of maternity care, accessibility and delivery of services, and clinical disparities impacting Indigenous maternal health within Canada. It also identifies current knowledge gaps in research on these topics.

**Methods:**

A scoping review was completed using the Preferred Reporting Items for Systematic Reviews and Meta-Analyses (PRISMA) statement guidelines and the extension for scoping reviews. The search for relevant papers was performed in PubMed, CINAHL, and SCOPUS electronic databases and included any empirical literature written in English and published during 2006 – 2021. The research team inductively coded 5 articles to develop a coding scheme, which was then applied to the remaining articles.

**Results:**

A total of 89 articles were included in the review, of which 32 were qualitative papers, 40 quantitative, 8 were mixed-methods publications, and 9 were review papers. The analysis of the articles resulted in identifying a range of overarching themes pertaining to the maternal health of Indigenous women within Canada including provision of services, clinical issues, education, health disparities, organization, spatial context, and impact of informal support. The results suggest that physical, psychological, organizational, and systemic barriers inhibit the quality-of-care pregnant Indigenous women receive, and that maternal health services are not consistently provided in a culturally safe manner. Results also suggest that, compared to non-Indigenous pregnant women, Indigenous women are more likely to develop clinical complications during pregnancy, reflecting the structural impacts of colonization that continue to negatively influence Indigenous maternal health and well-being.

**Conclusions:**

There are many complex barriers that prevent Indigenous women from receiving high quality and culturally appropriate maternal care. Possible areas that could address the service gaps illuminated through this review include the implementation of cultural considerations across health care jurisdictions within Canada.

**Supplementary Information:**

The online version contains supplementary material available at 10.1186/s12884-023-05645-y.

## Background

Maternal health care plays a key role in ensuring the growth and development of the unborn child and is also necessary to protect the health and well-being of the mother [1]. Despite strides made to improve maternal well-being globally and the World Health Organization’s (WHO) commitment to reducing maternal morbidity and mortality, disparities in health outcomes and access to services continue to exist. Internationally, Indigenous pregnant women^1^ tend to be at a heightened risk of experiencing complications throughout pregnancy, resulting in higher rates of maternal morbidity and mortality [1]. This increased level of risk is due to a range of complex determinants including the ongoing impacts of colonization and associated social inequalities resulting from dispossession of land and resources [2].

The need to address maternal health care globally has been supported by a range of international organizations and United Nations’ agencies. WHO states that reducing rates of maternal mortality should be prioritized on the global agenda and positioned improving maternal health as one of its key priorities [3]. To improve maternal health, WHO has established partnerships with Member States (see Appendix 1) with the common goal of addressing inequalities regarding the access and quality of maternal and child health services, as well as strengthening global health systems to collect precise data to address needs and priorities of women [3].

Inequities in the provision of maternal health care services are especially evident when comparing non-Indigenous and Indigenous populations residing both on- and off-reserve. Within Canada, examples of disparities, inequities, and inequalities are notably present among many domains within maternal health and the access and delivery of health services [4–21]. According to the Indian Act of 1876, it is the Canadian government's responsibility to provide health care to First Nations living on-reserve [22]. For the general population and Indigenous people living off-reserve, the responsibility of health care falls upon provincial and territorial governments [22]. Despite the responsibility placed on the federal and provincial governments to provide health care services to Indigenous population in Canada, it has not been adequately and consistently offered to Indigenous communities. Moreover, the Indian Act itself has been criticized as perpetuating health inequalities, and adding to racism and discrimination experienced by Indigenous individuals and families when accessing health care services throughout Canada [22].

Prior to colonization, Indigenous women gave birth in their communities with support from family, community, and local midwives [23]. Midwives supported pregnant and birthing mothers using culturally centred knowledge and practices [23]. The colonization of maternal care and birthing practices has resulted in the displacement of culturally important knowledge and its replacement with hard to access, and often suboptimal medicalized care within Indigenous communities [24]. Indigenous women residing on-reserve in remote settings are often forced to receive maternal health care and deliver their babies in faraway urban centres, which removes them from their family, friends, and community [24]. These evacuation processes negatively impact birth experiences due to the discrimination, racism and abuse frequently encountered [24]. Indigenous practices, knowledges and beliefs have also been ignored and disregarded by health providers [24]. Moreover, dispossession associated with the transfer of knowledge within Indigenous communities has also led to the loss of access to the support of Knowledge Holders such as Indigenous midwives and doulas, although recently there has been a revival of Indigenous birthing practices across a number of communities within Canada [23]. The National Aboriginal Council of Midwives (NACM) is one of the organizations helping to promote the rebuilding of Indigenous midwifery services among Indigenous communities [25].

In Canada, the literature commonly reports disparities in access to maternal health care between Indigenous and non-Indigenous women [4–21]. Indigenous pregnant women are disproportionately impacted by illnesses and diseases, for which preventative measures and treatments exist. However, access to these services is often denied to Indigenous women due to systemic oppression and racism institutionalized within the Canadian health services. This may explain why the literature tends to reports that Indigenous pregnant women experience mental health (anxiety and depression) challenges, along with increased rates of conditions such as Fetal alcohol spectrum disorder (FASD), human immunodeficiency virus (HIV), diabetes (pre-gestational, gestational, and postpartum diabetes), obesity, as well as increased environmental exposures and substance use (tobacco smoking, alcohol consumption, and drug use), heightened risk of experiencing maternal mortality, and increased occurrence of birth resulting in low-birth weight infants and stillbirths [4–12, 15–17, 26–33]. The literature reviewed was found to focus on the deficit-based aspects of maternal health. Indigenous pregnant women face direct and indirect barriers to accessing care, ranging from community-level complications surrounding geographical location and transportation, to issues related to federal or provincial jurisdictions [34]. The aforementioned health disparities are also the result of colonization, intergenerational trauma, sixties or millennium scoop, discrimination, abuse, residential schools, and the oppression of Indigenous communities [35].

While the literature identifies disparities in maternal health outcomes between Indigenous and non-Indigenous populations within Canada, and points out the inequities in access to maternal health care services, there have been limited attempts to synthesize this data and identify knowledge gaps. This, however, is an important step in recognizing ultimately how maternal health services offered to Indigenous mothers can be improved. The aim of this review is to address these gaps by examining the literature adhering to the following main and secondary research questions:

1. What is currently known in the empirical literature about the maternal health care disparities experienced by pregnant Indigenous women within Canada?
  a What are the factors causing or contributing to the maternal healthcare disparities experienced by Indigenous women?
  b How can the maternal health of pregnant Indigenous women be improved?
  c What kind of access do pregnant Indigenous women have to preventive care and treatment that address the health problems experienced?

## Methods

A scoping review was completed following Arksey and O’Malley [36] methodology using the Preferred Reporting Items for Systematic Reviews and Meta-Analyses (PRISMA) guidelines and the extension for scoping reviews [37]. Arksey and O’Malley recommend using five steps when conducting scoping reviews: identifying the research question, identifying relevant studies, study selection, data extraction / charting the data, and summarizing and reporting the results [36].

**Fig. 1:**
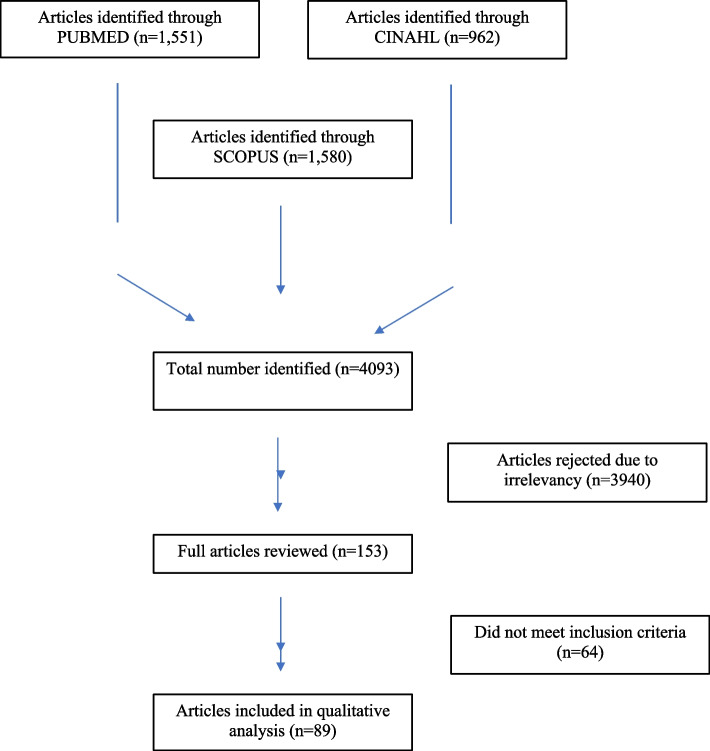
The PRISMA Extension for Scoping Reviews (PRISMA-ScR) flow diagram represents the formal literature review and screening process developed for the scoping review

When identifying the research question, four authors (MB, NH, EN, ZA) worked in close collaboration to determine the research question for this scoping review. The authors developed the following primary research question: What is currently known in the empirical literature about the maternal health care disparities experienced by pregnant Indigenous women within Canada? The search for relevant papers was performed in PubMed, CINAHL, and SCOPUS electronic databases and included any empirical literature published during 2006 – 2021. The search strategies were drafted by two members of the research team (ZA, MB) with the assistance of an academic librarian and refined through discussions with the research team. The final search results were exported into COVIDENCE for sorting and analysis [38]. The search terms included four concepts including Indigenous, clinical maternal health, Canada, and access to health services. When selecting studies, articles were included in the review according to the following criteria: 1) peer-reviewed and empirical papers; 2) published within set time parameters; 3) included a focus on Canada; 4) published in English, and 5) focused on pregnant Indigenous women and maternal health services. The PRISMA Extension for Scoping Reviews (PRISMA-ScR) flow diagram represents the formal literature review and screening process developed (see Fig. 1). A total of 5,834 articles were identified in the search strategy. After removing duplicates, the remaining 4,093 articles were screened by title, and abstract, and 3,940 articles were then considered irrelevant. The full texts of 153 articles were assessed for eligibility. Of these, 64 were excluded, as they did not meet the inclusion criteria.

To chart the data obtained, an Excel document was created as a literature extraction tool and used to record each article’s general information (author, title, journal publication year), along with study objectives, methods, and location. The articles included in the scoping review were also analyzed thematically. Each author independently read five articles and inductively identified key themes present in the literature. During the coding process, some articles were double coded as the content was relevant for two or more established themes. While themes were inductively derived from the data, the authors’ commitment to the recognition of the impact of colonization led to the examination of the presence of this topic in the selected articles. This information was recorded in order to determine how many of the included articles discussed the impact of colonization on the health and well-being of Indigenous Peoples within Canada. As such, the number of articles that referred to the impacts of colonization on the health and well-being of Indigenous women were highlighted in this review.

## Results

### Characteristics of included studies

**Fig. 2:**
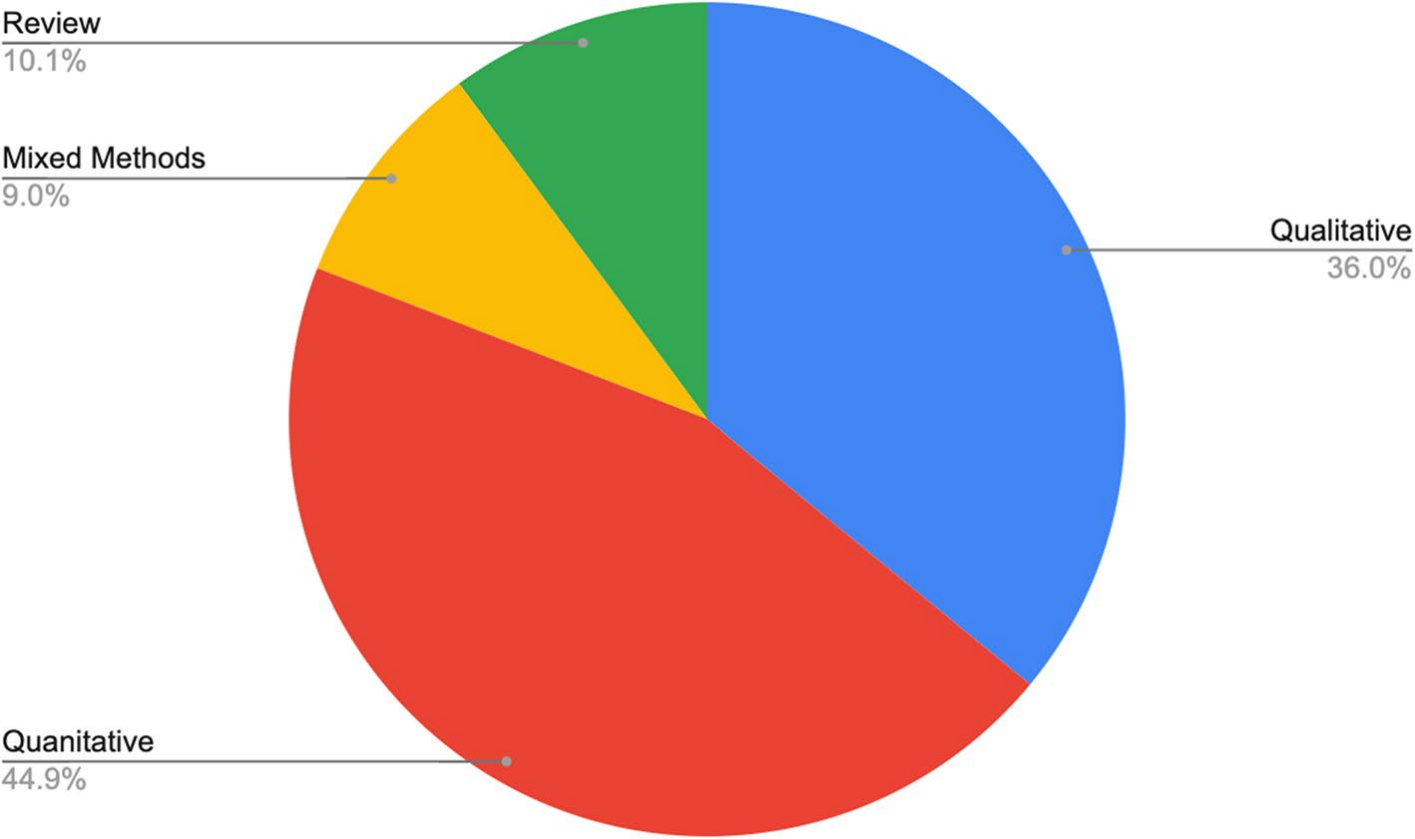
Number of articles that used qualitative, quantitative, and mixed-methods research designs

**Fig. 3:**
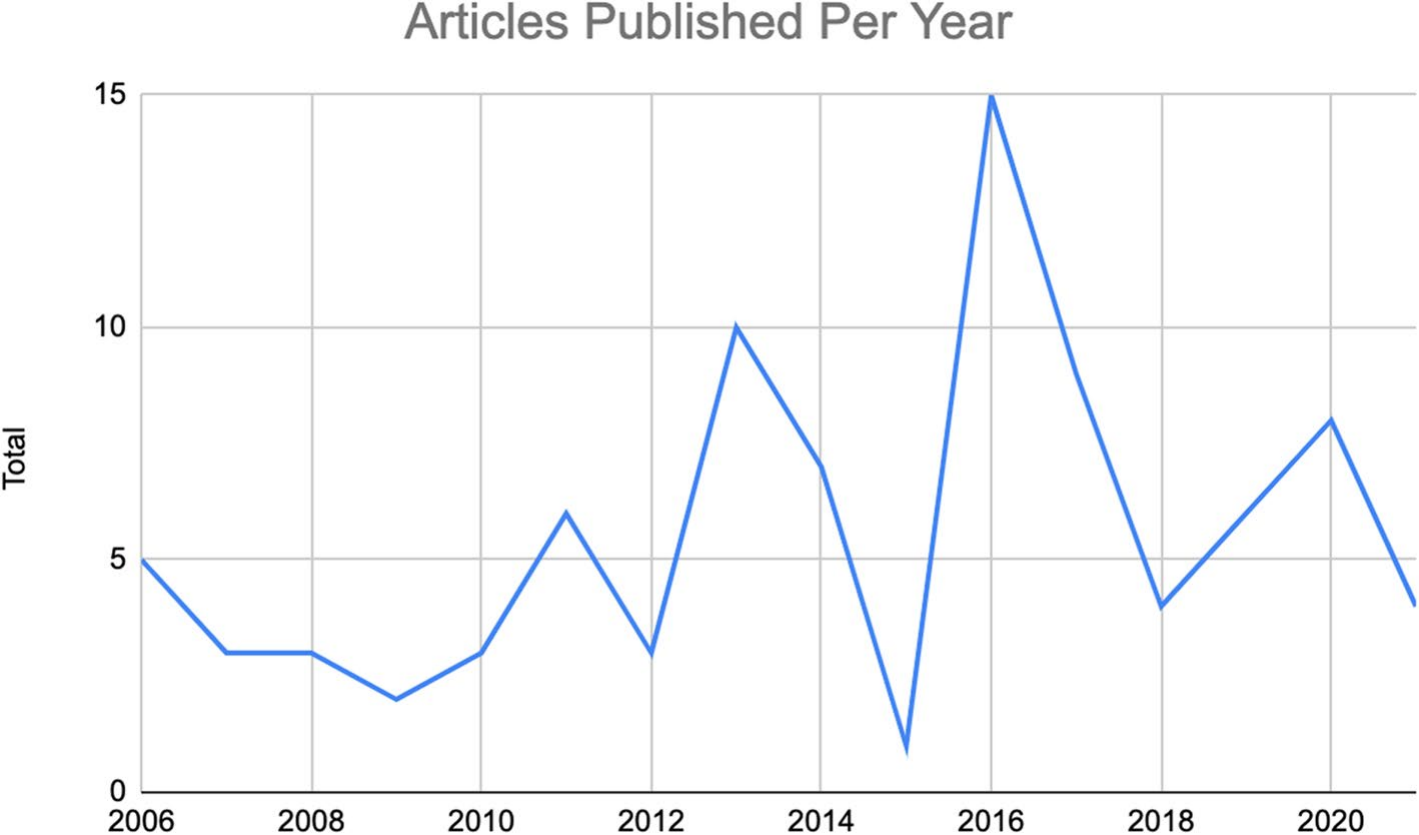
Number of articles published per year from 2006 to 2021

In total, 89 articles met the inclusion criteria out of the 4,093 articles identified (Fig. 1). Among them, 32 papers were qualitative, 40 quantitative, 9 review papers, with only 8 mixed-methods publications (see Fig. 2). Figure 3 summarizes the distribution of the published literature by the year of publication. Key themes noted from the literature include: Health Disparities; Provision of Services, Education, Resources and Quality of Care; Spatial Context; Informal Support; and Organization of Care. The prevalence of each theme is summarized in Fig. 3. In what follows, we provide more details about the thematic analysis.

### Health disparities

In this thematic category, we coded 27 papers that focused on health inequities among Indigenous and non-Indigenous pregnant women within Canada. A large number of articles discussed the topic of prenatal weight gain, finding that Indigenous women tended to gain more than the recommended weight during pregnancy [18, 21, 39–41]. In addition, diabetes was a commonly discussed topic, with many of the articles concluding rates of pre-gestational diabetes mellitus (pre-GDM) and gestational diabetes mellitus (GDM) are higher for Indigenous women compared to the general population of Canadian women [5, 6, 11, 14–17, 20, 29]. Indigenous women were found to be more likely to develop GDM and pre-GDM [5, 6, 11, 15]. With the exception of one study by Riddell and colleagues (2016) which indicated that after taking into consideration the age of participants, the occurrence of GDM was similar between Indigenous women and non-Indigenous women [42].

The next common topic reported in the literature was that Indigenous women living both on and off-reserve reported experiencing depression, anxiety, thoughts surrounding self-harm, and inadequate social support at higher rates compared to non-Indigenous women [13, 21, 27, 28, 31]. However, Bowen and colleagues (2009) reported that while Indigenous women were more likely to suffer from depression during pregnancy and postpartum, this finding was not significantly higher compared to non-Indigenous women [27].

In addition to mental health, social support, weight gain, and diabetes, the literature also focused on the topic of nutrition and the impact of contaminants within foods and the local environment on maternal health of Indigenous women. The local environment, tradition, and culture play a key role in what foods are available and consumed by Indigenous people living on reserves [10]. For example, among the Cree First Nations of Eeyou Istchee in northern Quebec, a traditionally consumed and hunted food is local fish [10]. However, the consumption of fish was regarded as a health concern in this community, as the fish in the local environment was found to contain high levels of Hg [10]. In a study published by Ripley et al., the toxins present in the fish were seen to pass along to the Cree community, with blood/hair Hg levels higher in Cree women compared to other non-Indigenous women within Canada [10]. A similar finding was reported in another study where the level of Hg was found to be 18 times higher among Nunavummiut pregnant women, compared to pregnant women from Southern Quebec, while the presence of PCB was 3 times higher [9]. The topic of nutrition was only discussed in a few studies. Most notable, one study conducted in Saskatchewan discussed the occurrence of vitamin D3 insufficiencies, noting that 75.3% of First Nations pregnant women had vitamin D3 levels that were labeled insufficient [12].

Overall, the literature outlined a variety of health-related issues such as younger maternal age, substance use, weight gain, diabetes, depression, and vitamin deficiencies [4–6, 11–13, 15, 18, 19, 21, 27, 28, 31, 42]. Some papers also focused on the presence of Mercury (Hg) and Polychlorinated biphenyls (PCBs) and their impacts on maternal health care outcomes [9, 10, 12]. Socio-economic factors, experiences of violence, and unemployment rates were also cited as major causes for health disparities by some authors [4–21].

### Provision of services

**Fig. 4:**
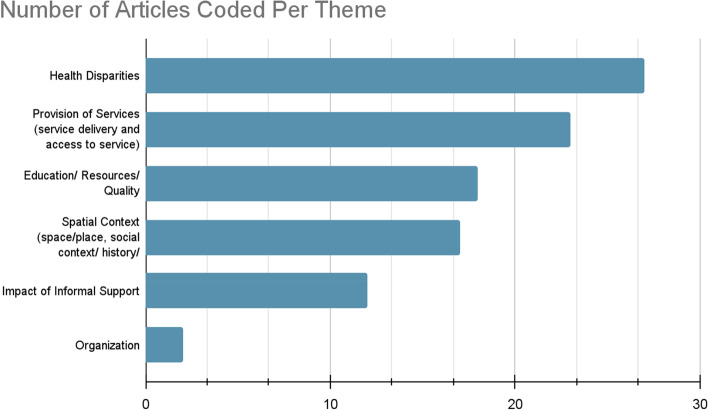
Number of articles coded for each theme

In this second largest category, 23 articles were coded. We included papers that examined access to maternal care services (*n* = 14) and those that focused on various aspects of service delivery (*n* = 9) (see Fig. 4). Service delivery identifies how health services and patient care are being provided. The care provided to Indigenous women was most commonly described by Indigenous mothers as a negative interaction [39, 43–47]. Women reported positive interactions with the health system and providers when they received context-specific care that included supporting Indigenous mothers to receive care on a walk-in basis [44, 48]. Moreover, context-specific care encourages collaboration with Indigenous women and community members in the creation and delivery of health programs [48].

The majority of the articles in this category stated that Indigenous women were not comfortable with the maternal health care provided by health professionals [39, 43–47]. One of the recurring topics was the concern that health services were not being provided in a culturally safe manner. Participants felt that the current health system is rigid and not flexible with their schedules [43, 44]. Health facilities typically provide care based on specific appointments that tend to be inflexible and have implemented policies that do not meet the needs of Indigenous women [44]. For instance, one study conducted near Ottawa, Ontario found that health professionals advising Indigenous women on prenatal weight gain would provide care often ridden with shame and blame, indicating that health providers do not consistently provide culturally safe care [39].

Indigenous women utilizing health services also expressed feeling disrespected and shared experiences of racism and stigma. Many felt that their health concerns were disregarded by health care providers [45–47]. Moreover, Indigenous women were less likely to utilize health services offered by government- run programs or facilities. This was displayed in a study published by Abdullah and colleagues (2017), who found that Indigenous women were less likely to use medical services offered through government health systems because of decreased standards of medical care, decreased specialist referral, reduced access to higher quality medical therapies, and social inequalities [34].

To combat these circumstances, an alternative format of care was presented by some authors. The adapted format of care would provide empowering and flexible care received on a walk-in basis [44]. Indigenous women found this format to be more flexible, accommodating, and culturally safe [44]. Participants indicated that providing context-specific care can help mitigate some of the barriers to care specific to Indigenous women [44]. For example, primary health models of care that were developed collaboratively with Indigenous communities to offer programs and coordinate access to culturally appropriate and sensitive care were shown to result in more positive maternal health care outcomes and stronger patient-provider relationships [48].

Another important topic that was identified during the analysis was access to services and, specifically, availability of care and how health services were being utilized. The main barriers to accessing services included geographical location, diagnosis of diabetes, and experiences of stigma and discrimination [14, 20, 47–51]. Efforts to increase access to health services, such as remote offerings of health resources, has shown great promise in promoting utilization of care [52]. For example, a study conducted by Hui et al. (2021) suggests that providing maternal health care education remotely increased program participation among Indigenous pregnant women in rural and remote locations in Manitoba [52]. The program included a maternal health care chat group and community support group where participants could connect with each other and discuss their concerns and receive advice [52]. Providing a remote program offering increased participation from 36 to 54% within the first year of implementation [52].

Studies found that a commonly reported reason for the lack of utilization of maternal health care services among Indigenous women was the participant's geographical location. Women experienced transportation issues and fewer options in terms of location and the type of maternal health care locally available [20, 47, 48, 51]. The availability of transportation played a key role in determining if the health services would be accessible [48]. This is problematic, as throughout the course of pregnancy, the WHO prenatal care model recommends 8 visits between the women and their health provider to receive maternal health care [48]. The issue of care availability, transportation, and accessibility becomes increasingly complicated when additional clinical issues are present, as typically, health provider visit frequency increases with additional diagnoses, such as diabetes [48]. In addition, Indigenous women reported feeling frustrated and powerless in situations where they were unable to have a choice in who provides their medical care [47]. A number of articles discussed the impact of care provided when a diagnosis of GDM was made [14, 20, 49]. Within these studies, even though GDM rates tend to be higher among Indigenous women, Indigenous women with GDM were found to receive fewer postpartum oral glucose tolerance tests and had reduced rates of health service utilization during pregnancy and postpartum [14, 20].

Providing transportation to attend medical appointments is not always a clear-cut solution to increase access to services. When transportation is provided, Indigenous women continue to face barriers including a lack of available drivers, resulting in issues with scheduling appointments [48]. While most studies discussing transportation as a barrier focused on the access of care from the perspective of a patient, one study focused on how health providers expressed difficulty providing care due to geographic location. In this study, the staff mentioned that there are many barriers to providing health services in rural communities, and one of the biggest challenges is to travel long distances to provide care [51]. The provider mentioned that it is also a commonly cited issue for clients who may be unable to access the services due to transportation issues [51].

### Education, resources and improving the quality of care

Among the included publications, 18 articles were coded under this theme, which focused on how Indigenous women receive education about maternal health, the various educational resources available to them, and the quality of care provided to them. Three studies reported that providing education and resources to Indigenous pregnant women improved their health and increased engagement in health management [53–55]. Moreover, health education programs offered to health providers were found to help improve the quality of care provided to Indigenous women [56]. However, providing education and resources in a negative and discriminatory way can result in negative impacts on health and well-being [46]. Furthermore, while providing educational sessions to health providers did appear to have a positive impact, it is imperative that additional measures, such as routine programming, are implemented due to high staff turnover and shortages [56].

Providing resources and educational programs to Indigenous mothers has been found to help improve their health and well-being, and increase engagement in health management programs [53–55]. Programs and resources that are provided on a no-condition basis have been seen to be successful and help promote health among Indigenous pregnant women and mothers [53]. Previously implemented health programs and benefits were seen to put Indigenous pregnant women at a lower risk of experiencing low birth weight, preterm birth, decrease drug use by time of delivery, and had a higher rate of initiating breastfeeding [53]. While providing educational services and resources are seen to increase positive health outcomes, it has also been recommended that the development of resources for Indigenous women should be done with their full participation and consultation [55, 57]. Indigenous maternal health programs should incorporate community involvement, such as community health workers, female relatives, and Elders [57]. To improve dietary counselling and education, food suggestions should be provided with an emphasis on cultural learning, and the consideration of incorporating traditional foods [57].

A number of articles also outlined how educational services have previously created resources and materials that were inappropriate and discriminatory towards Indigenous pregnant women [46, 51, 58]. Language used within educational programming has placed blame on Indigenous women for their behaviour during pregnancy [51]. When discussing sensitive topics such as substance and alcohol use during pregnancy, it is important for health program developers to take into consideration the complex factors that contribute to smoking, drinking, and drug use during pregnancy [51]. If these considerations are not discussed and accounted for, health programming or resources may result in causing more harm and further marginalizing Indigenous women [51].

While educational programming has been seen to provide some beneficial outcomes to Indigenous pregnant women, the results also illustrated that this type of programming could be taught to health providers to improve the quality of care [56]. For example, a study looking at the chart audits of patients with GDM at a health clinic in a northern community revealed that the general screening provided was not consistent with the standard of care [56]. The sample group for this study included 33 patients, all of which received a blood glucose test during pregnancy [56]. However, the type of test used, and the timing of the test being ordered varied significantly [56]. An educational session was implemented to address the variations of care being provided, and the study found that while the sessions brought awareness to the issue, adherence to the recommendations was lacking due to high staff turnover and shortages [56]. This situation highlighted the importance of ensuring that routine programming is offered to not only physicians, but to all individuals providing care within the health system [56]. One study recommended that health programming and services need to increase culturally appropriate intergenerational education and promote the learning of cultural ideas systems and emotional lives of patients among health providers [57].

### Spatial context

Spatial context is a broad theme that includes the influences and impacts of history, culture, and surrounding physical and spiritual environments on beliefs, behaviours, and ideologies. A total of 17 published studies discussed topics related to this theme. Out of the 17 coded, 7 articles were further categorized under the sub-theme Space and Place, while 10 discussed the spatial context. Space and place incorporate the connections Indigenous Peoples and communities have with the physical environment and the important role of these relationships for Indigenous identity and practice. These articles discussed the importance of access to Indigenous foods, and the role that the ecology and physical environment plays within Indigenous culture and knowledge systems [59, 60]. The articles coded under this theme primarily discussed how the winter and summer months presented barriers in accessing traditional foods and engaging in physical activity.

The incorporation of Indigenous foods during pregnancy has been associated with improved nutrition and promotion of cultural values [60], however, gaining access to these locally harvested foods presented challenges for some participants of studies included in this scoping review [40, 60]. Indigenous pregnant women living in remote communities had challenges accessing food throughout their pregnancy, especially during the winter months [60]. In the winter, food quickly spoiled, was elevated in price, and Indigenous women faced transportation barriers associated with the weather [60]. Fast food options impaired the participants’ ability to pick healthier meal options [40]. The weather in some of the locations was also mentioned to play a role in whether or not the participants were able to engage in physical activity during pregnancy [40]. Both winter and summer seasons presented challenges associated with the temperatures that discouraged outdoor physical activity [40].

The second sub-theme, historical and continued colonization focuses on these processes and their impacts on the relationship between Indigenous communities and the Canadian healthcare system. Articles that provide historical context and insight into the influence’s colonization has on health outcomes and/or access to health care services indicate that these structural determinants play a significant role in the health and well-being of Indigenous women within Canada [19, 21, 45, 61–65]. A number of articles mentioned the impact of intergenerational trauma, residential schools, racism, and discrimination negatively shaped access to health care services for Indigenous mothers [21, 45, 66].

Many studies emphasized that it is important to review the lived experiences of Indigenous women to contextualize health disparities and inequalities [19, 21, 45, 61–65]. Substance use is related to many factors including stress, context, isolation, general health, age, genetics, resilience, cultural discrimination, experiences with violence, access to maternal health care, social policy, and poverty [61]. Indigenous women’s perceptions of wellness are also impacted by a range of distal factors such as trauma, abuse, and violence [63]. Previous traumatic experiences provide context on substance abuse while culture and spirituality play a significant role in the perceptions of overall health and well-being [63]. Western ideologies can be harmful to self-perceptions [41]. Moreover, the attempted erasure of Indigenous culture and traditional practices due to colonial policy and practice has disrupted hunting and gathering practices, language and intergenerational knowledge surrounding maternal care and birth [21, 41, 67].

The history of colonization and discrimination directed towards Indigenous Peoples has elicited fear or a lack of trust when accessing government services in the health system. Indigenous women have reported that health service providers did not respect their identity and they did not feel safe in accessing care [66]. This was strongly displayed in one study where a participant indicated that child protection services would regularly be called when Indigenous mothers would be giving birth [21].

### Informal support

Out of the included publications, 12 were coded under this theme. Family, friends, peers, Elders, and community members often provide informal support during the prenatal period to Indigenous mothers. In a number of studies, social support was found to improve or discourage the participation in physical activity during pregnancy, the occurrence of weight gain, and nutritional choices [40, 58, 67–71]. Social support was also found to help or hinder a woman's management of gestational and type two diabetes [72].

The literature discussed the positive impact of social support, and how social networks support resilience, promote feelings of connectedness with the community, and reduce experiences of stress [69]. Articles emphasized the importance of family, community, and friends, noting that pregnant women would turn to their significant others for personal advice, medical guidance, and support during pregnancy [48]. While overall, social support had a positive impact, one researcher pointed out how negative social support reduced mothers’ ability to engage in healthy behaviours that can prevent weight gain and diabetes [40]. For example, Indigenous mothers who gained beyond the recommended weight during pregnancy shared that while they understood that junk food should be avoided during pregnancy, it was hard to do so because their social environment (friends and family) continued to engage in unhealthy habits [40]. In the study conducted by Black and Colleagues (2008), the researchers indicate that women who gained an appropriate amount of weight during their pregnancy reported having fewer negative influences that impacted their eating habits, compared to the women who gained beyond the recommended amount of weight [40].

Intergenerational knowledge sharing was commonly discussed throughout the articles included within this scoping review. Intergenerational knowledge plays a very significant role in the process of information sharing about nutrition, lifestyle, healthy behaviours and habits during pregnancy [48, 63, 64, 70, 71].

### Organization of care

The theme of organization of care included papers that examined the coordination of maternal health care services at an organizational or government levels, including federal, provincial, or municipal governments, non-governmental organizations, on-reserve, band-operated health services or not-for-profit organizations. Only 2 publications were coded under this theme.

A study published by Corcoran and Colleagues (2017), discussed the jurisdictions of care, as well as the federal, provincial, and territorial boundaries associated with the delivery of health services in Manitoba [34]. In the location of the study, the federal government funds health care in First Nation communities and for the transportation of women to birthing facilities in southern urban centres [34]. While the health facilities are federally funded, the health workers are provincially funded, managed and hired [34]. As such, the provincial health workers described barriers they encountered when trying to provide services to Indigenous women in federally operated health facilities. The health workers in the study noted that due to jurisdictional complications, they were unable to access patient records, resulting in an inability to efficiently provide care or order lab work [34].

Smoking cessation and the resources, policies, and funding associated with tackling this concern were discussed in another article coded in this thematic category. This study emphasized that when addressing tobacco smoking cessation, there needs to be a focus on creating a provincial smoking cessation strategy [73]. A study completed by Borland and Colleagues (2013), examined the quality of smoking cessation support available for pregnant and postpartum women in Ontario, Canada [73]. Within this study, the researchers indicated that the policy surrounding smoking is vague, there is a lack of available sustainable funding dedicated to cessation services, and a lack of engagement with exchanging knowledge surrounding smoking cessation practices [73]. To address these issues, the researchers recommend that the province incorporate a number of improvements, including but not limited to, a detailed smoking cessation strategy, integrated tobacco policy, increased taxation of tobacco, and increased sustainable funding towards resources to assist with smoking cessation [73].

## Discussion

The goal of this scoping review was to study Indigenous maternal health and understand what is currently known in the empirical literature, examine the access pregnant Indigenous women have to preventative care and treatment, highlight the factors causing or contributing to maternal healthcare disparities, and identify how the maternal health of Indigenous women can be improved. By providing an overview of these patterns and themes, this review aimed to address the knowledge gap in the literature while offering insight on the limitations of the Canadian healthcare system related to service delivery, access to services, quality of care, educational resources, health disparities, and acknowledgement and consideration of Indigenous knowledges and cultures.

According to the results of this review, the empirical literature suggests that the Canadian healthcare system is not consistently providing adequate care and support to Indigenous mothers across federal, provincial, and territorial jurisdictions. The results also suggest that many health services provided to Indigenous pregnant women often lack cultural sensitivity and are not culturally safe, which results in decreased utilization of health services [39, 43–47]. However, it is important to note that there has been evidence to suggest that providing context-specific care to Indigenous women has helped to alleviate some barriers to maternal care [44, 48]. Regarding the access of services, the results highlighted that geographical barriers can be overcome using new tools, such as remote maternal health care education programs. These programs can be delivered via online platforms and TV or radio broadcast, with the latter being leveraged in communities without reliable access to internet [52]. The review also pointed out that some educational resources targeting Indigenous pregnant women can utilize discriminatory language, and thus cause more harm than good [51, 58]. Additionally, the results suggest that educational programming should be made available not only to pregnant Indigenous women but also to healthcare providers and government officials. This may help to create more understanding, inclusive, and supportive environments.

The results of this scoping review highlight that the current policies and processes in place to dictate the provision of Indigenous health care and jurisdictional responsibilities are inadequate and harmful. The method in which federal, provincial, and territorial governments engage in service delivery feeds into the development of gaps in the provision of care [34]. For example, this review showed that the requirement to leave their place of residency to access maternal health care services restricts the connection with the land and disrupts cultural continuity [74]. Having access to the land helps enforce the connection to cultural supports and in turn, helps improve spiritual wellness. To combat the issues surrounding the provision of services, there must be an emphasis placed on providing care on-reserves and in a culturally appropriate manner [45–47].

The reviewed literature that included discussions surrounding contextual factors emphasized the negative impact of colonization on health. Laws such as the Indian Act impacted Indigenous Peoples’ connection to the land and cultural practices; displaced people from traditional territory, which in turn influenced cultural practices and resulted in the reduction of intergenerational knowledge regarding hunting, gathering foods, medicines, languages, teachings, and practices surrounding a healthy pregnancy and care for mother and child. The reviewed papers highlighted the importance of family, friends, and the community in shaping pregnant Indigenous women's maternal health outcomes. Having strong social support helps promote intergenerational knowledge transmission, promote healthy behaviours during pregnancy and impact maternal health outcomes [48, 63, 64, 68, 71].

Numerous striking health disparities were witnessed throughout the included articles pertaining to physical and mental health of Indigenous pregnant women [8, 13, 18, 21, 27, 28, 31, 39, 41, 51]. It is important to highlight these disparities and take them into account when conducting future research and administering health services to ensure that the differing needs of Indigenous pregnant women are accounted for and that these women receive high quality care pertaining to these needs.

### Implications for practice and research

It is important that the health system itself be flexible and accommodating to Indigenous patients as there are many geographic, social, and historical barriers that limit the ability and desire to access care [56]. Moreover, the results have shown the need to teach health providers how to administer care in a culturally appropriate manner [57]. However, educational programming should be done in an ongoing manner to ensure new staff that are hired as a result of employee turnover are properly trained [56]. There is also a need for additional educational programming tailored towards Indigenous pregnant women about the clinical issues they may face during pregnancy. However, the educational materials need to represent Indigenous pregnancies in a positive context, as this has not been the common practice [57, 58]. Educational resources and materials have been harmful and discriminatory towards Indigenous pregnant women. The results from this review recommend that educational resources and materials should be created with the input and participation of Indigenous women, and in doing so, more positive health outcomes may be achieved [51, 58]. Future research could involve having Indigenous women create educational material outlining the information they would like to know about pregnancy and how they would like to be taught the material. For example, one area where this is a possibility of improvement is educational programming surrounding foods. The programming must be done in a method that respects and promotes the consumption of traditional foods where accessible and available [57]. Overall, future research should move away from deficit-based research to address more of the distal determinants of Indigenous women’s health.

Furthermore, there needs to be action taken to address the issues surrounding jurisdictional gaps in the provision of care [34]. When creating policy regarding provision of care and allocation of resources for Indigenous pregnant women, it’s imperative that policy makers take into consideration the impact of colonization and racism in current exciting policies and systems. In addition, maternal health care and birthing centres need to be more geographically accessible to Indigenous pregnant women. This can be addressed by allocating resources among areas that have a reduced number of health facilities established. Having care close to home is crucial as it helps improve the women’s ability to access support from community based social networks.

Future areas of research need to include a more in-depth examination of the jurisdictional responsibilities to provide maternal health care to Indigenous women and their families living on and off reserve. The results from this scoping review highlight that current policies are flawed. Further research should be conducted to examine how policies surrounding Indigenous maternal health care can incorporate Indigenous women’s experiences and include them within policy reform.

### Strengths and limitations

A limitation of this scoping review is that it focused on academic literature and did not include grey literature in the search strategy. This is a limitation as information listed in grey literature could also provide insight into the topic of Indigenous maternal health. Moreover, the scoping review only included articles that were written in English, as such articles written in French and other languages were not included. In addition, the scoping review only reviewed articles that spoke to the Canadian context because of the authors focus on the government and administration structure, but some Indigenous Peoples may be travelling and moving across international borders. This scoping review also only included articles published after 2006. As such, there could be topics and relevant data missing in the results. In addition, being consistent with scoping review methodology, the papers were reviewed, however the researchers did not comment on the quality. Notwithstanding these limitations, the strengths of this review include that the results assist in adding to the academic literature and fills the current research gap surrounding a lack of available synthetized reviews examining this topic. Moreover, this scoping review provides a unique in-depth overview of the maternal health issues being experienced, perspectives from Indigenous women and health providers, along with an examination of the organization of care, impact of spatial context, and the impacts of informal social and cultural supports. The content within this scoping review helps provide readers with an overview on the current state of Indigenous maternal health within Canada and highlights areas for improvements, while providing recommendations.

## Conclusions

The current state of Indigenous maternal health within Canada clearly needs to be improved. Providing high-quality care that is culturally appropriate and respectful is one avenue that could start to bridge the many gaps present in the care provided to and disparities in health outcomes experienced by Indigenous women compared to non-Indigenous women. When care programs are created, the consideration of context and previous experiences need to be taken into account. Indigenous community leaders and members should be included in the planning of health initiatives to ensure that programming is respectful and incorporates Indigenous healing practices [56]. Health centers providing maternal care need to be accommodating to Indigenous women and understand that strict, appointment-only facilities add additional barriers in accessing services. Studies included within this scoping review have reported that flexible scheduling can support service access for women who experience transportation issues and time constraints [48]. This also highlighted the need for the organization of care and provision of services offering maternal health care to Indigenous women to be improved. Jurisdictional policy confusion causes gaps in provision of care and unnecessary barriers to providing care [34]. Governments at federal, provincial, and territorial levels need to collaborate to create policies that do not elicit confusion and create further barriers for health facilities or providers that deliver maternal services to Indigenous women.

## Supplementary Information

**Additional file 1:** **Appendix 1.** Member states of the WHO.

**Additional file 2.** Characteristics of the studies included in the scoping review.

## Data Availability

All data generated or analysed during this study are included in this published article and its supplementary information files.

## Abbreviations

WHO: World Health Organization
NACM: The National Aboriginal Council of Midwives
FASD: Fetal Alcohol Spectrum Disorder
HIV: Human immunodeficiency virus
Hg: Mercury
PCB: Polychlorinated Biphenyls
PRISMA: Preferred Reporting Items for Systematic Reviews and Meta-Analyses
pre-GDM: Pre-gestational diabetes mellitus
GDM: Gestational Diabetes Mellitus

## Footnotes

1 While the term women has been used throughout this paper, the authors recognize that not all people who can get pregnant identify as women.
