# Supplementary material for: Indigenous maternal health and health services within Canada: a scoping review
Source: BMC Pregnancy Childbirth. 2023 May 8;23:327. doi: 10.1186/s12884-023-05645-y (PMC10165845; doi:10.1186/s12884-023-05645-y)
Supplement: Supplementary file 1 — Additional file 1: Appendix 1. Member states of the WHO. [file 12884_2023_5645_MOESM1_ESM.docx]

Appendix 1: Member states of the WHO

OECD. International Regulatory Co-operation. The Role of International Organisations in Fostering Better Rules of Globalisation. 2016;

| Afghanistan | Albania | Algeria | Andorra | Angola | Antigua and Barbuda |
| --- | --- | --- | --- | --- | --- |
| Argentina | Armenia | Australia | Austria | Azerbaijan | Bahamas |
| Bahrain | Bangladesh | Barbados | Belarus | Belgium | Belize |
| Benin | Bhutan | Bolivia | Bosnia and Herzegovina | Botswana | Brazil |
| Brunei Darussalam | Bulgaria | Burkina Faso | Burundi | Cabo Verde | Cambodia |
| Cameroon | Canada | Central African Republic | Chad | Chile | China (People’s Republic of) |
| Columbia | Comoros | Congo | Cook Island | Costa Rica | Côte d'Ivoire |
| Croatia | Cuba | Cyprus | Czech Republic | Democratic People’s Republic of Korea | Democratic Republic of the Congo |
| Denmark | Djibouti | Dominica | Dominican Republic | Ecuador | Egypt |
| El Salvador | Equatorial Guinea | Eritrea | Estonia | Ethiopia | Fiji |
| Finland | Former Yugoslav Republic of Macedonia | France | Gabon | Gambia | Georgia |
| Germany | Ghana | Greece | Granada | Guatemala | Guinea |
| Guinea-Bissau | Guyana | Haiti | Honduras | Hungary | Iceland |
| India | Indonesia | Iran | Iraq | Ireland | Israel |
| Italy | Jamaica | Japan | Jordan | Kazakhstan | Kenya |
| Kiribati | Korea | Kuwait | Kyrgyzstan | Lao People’s democratic republic | Latvia |
| Lebanon | Lesotho | Liberia | Libya | Lithuania | Luxembourg |
| Madagascar | Malawi | Malaysia | Maldives | Mali | Malta |
| Marshall Islands | Mauritania | Mauritius | Mexico | Micronesia | Moldova |
| Monaco | Mongolia | Montenegro | Morocco | Mozambique | Myanmar |
| Namibia | Nauru | Nepal | Netherlands | New Zealand | Nicaragua |
| Republic of Niger | Nigeria | Niue | Norway | Oman | Pakistan |
| Palau | Panama | Papua | New Guinea | Paraguay | Peru |
| Philippines | Poland | Portugal | Qatar | Romania | Russia |
| Rwanda | Saint Kitts and Nevis | Saint Lucia | Saint Vincent and the Grenadines | Samoa | San Marino |
| Sao Tome and Principe | Saudi Arabia | Senegal | Serbia | Seychelles | Sierra Leone |
| Singapore | Slovakia | Slovenia | Solomon Island | Somalia | South Africa |
| South Sudan | Spain | Sri Lanka | Sudan | Suriname | Swaziland |
| Sweden | Switzerland | Syrian Arab Republic | Tajikistan | Tanzania | Thailand |
| Timor-Leste | Togo | Tonga | Trinidad and Tobago | Tunisia | Turkey |
| Turkmenistan | Tuvalu | Uganda | Ukraine | United Arab Emirates | United Kingdom |
| United States | Uruguay | Uzbekistan | Vanuatu | Venezuela | Viet Nam |
| Yemen | Zambia | Zimbabwe |  |  |  |
